# Analyses of the Bacterial Contamination on Belgian Broiler Carcasses at Retail Level

**DOI:** 10.3389/fmicb.2020.539540

**Published:** 2020-09-16

**Authors:** Zhongjia Yu, Marie Joossens, Kurt Houf

**Affiliations:** ^1^ Department of Veterinary Public Health and Food Safety, Faculty of Veterinary Medicine, Ghent University, Merelbeke, Belgium; ^2^ Laboratory of Microbiology, Department of Biochemistry and Microbiology, Ghent University, Ghent, Belgium

**Keywords:** broiler carcass, bacterial distribution, retail, MALDI-TOF MS, 16S rRNA amplicon sequencing

## Abstract

Broilers are not equally exposed to bacterial contamination during rearing and processing, resulting in areas with different bacterial communities on carcasses at retail. The determination of these communities is also affected by the examination methods applied. The present study aimed to assess the bacterial communities on neck, breast, and back skin on broiler carcasses at retail through classical International Organization of Standardization based isolation methods combined with identification by matrix-assisted laser desorption ionization time-of-flight mass spectrum (MALDI-TOF MS) and 16S amplicon sequencing. Twelve commercially and eight organically reared broilers slaughtered in four slaughterhouses were examined. Significantly higher anaerobic bacterial counts were observed on the neck skin than on the breast and back skin. By the combination of cultivation and amplicon sequencing, remarkable shifts in bacterial communities were determined on the breast and back skin, but not on the neck skin. Although the aerobic bacteria contamination levels were not different between the areas, different bacterial communities were observed. The impact of the slaughterhouse to the overall microbial composition was rather small. Organically reared broilers had unique bacterial communities. In conclusion, compared to the breast skin, the neck, and back skin had a larger potential for bacterial spoilage, in particular when anaerobic storage conditions are applied. The distribution of bacteria on the different areas could be related to the contamination during slaughter as well as the bird-rearing methods.

## Introduction

Bacterial contamination of poultry products is the main cause of accelerated spoilage as well as a potential risk for foodborne infections. When broilers are processed in slaughterhouses and cutting plants, the skin and intestinal tract of the chickens, the slaughter and cutting equipment, and the environment and staff are all potential sources of direct or cross contamination (Surak, 2002). In particular, contact between carcasses and contaminated equipment has been identified as the major transmission route for foodborne pathogens, such as *Salmonella* and *Campylobacter* (Herman et al., 2002, 2003).

In most poultry slaughter facilities over the world, the slaughter processing line is designed as “head down position,” where chickens are hooked by their feet in the shackles on the conveyor belt during the entire process. In this way, neck skin collects most contamination due to the application of water in several slaughtering steps, such as defeathering and intermediate and final rinsing prior to chilling (Baré et al., 2013). The different areas on a broiler carcass are not equally exposed, resulting in areas with higher and lower bacterial contaminations. According to EU regulation EC 2073/2005, after chilling, the neck area is, however, the targeted sampling site for the detection of *Salmonella* (Cox et al., 2010). The back skin has been identified as the second most bacterial contaminated area due to potential contact with the intestinal content during the evisceration (Bodnaruk et al., 1998; Smith et al., 2007).

At present, comparisons of the bacterial load on different carcass areas including the bacterial diversity have not been reported. Furthermore, according to ISO 17604:2015, different sampling methods for the bacterial examination of poultry carcasses can be applied (ISO, 2015). One of the sampling methods often applied is whole carcass rinse sampling (Capita et al., 2004). However, with this method, no differentiation between carcass areas can be made, and the highest contaminated area dominates the final outcome. A majority of the carcasses are further processed into different cuts, and knowledge on bacterial contamination levels as well as diversity could result in a more differentiated predicted shelf life and contribute to reduce food loss.

The microbiome of a slaughterhouse environment consists of spoilage, pathogenic, and resident bacteria, and it contributes largely to a microbial shift on chicken meat (Tsola et al., 2008). At present, studies on the bacterial diversity on different areas on broiler carcasses, or on chicken products processed in different slaughterhouses, are scarce. This information is, however, a first step needed to assess the relation between microbiome, processing environment, and final product contamination.

For the determination of general bacterial parameters such as total aerobic bacteria (TAB), cultivation is still often performed using general media, though without detailed identifications. For the identification of bacteria, matrix-assisted laser desorption ionization time-of-flight mass spectrum (MALDI-TOF MS) has already become a rapid and efficient identification tool in clinical settings, and it finds it application in food microbiology (Höll et al., 2016). In the study of bacterial populations, both culture-dependent and culture-independent methods are applied. Culture-independent methods, like 16S rRNA gene amplicon sequencing, can generate in a feasible way more detailed microbiome profiles including those of uncultivable bacteria, but their application in food microbiology is still emerging. However, culture-independent data can be biased by several factors, such as sample storage, DNA-extraction method, choice of primers, sequencing technology, data analyses, and reference database. Therefore, combination of culture-dependent and culture-independent approaches could provide more detailed and accurate information (Lagier et al., 2016; Yu et al., 2019).

The aims of the present study are to determine the contamination levels as well as the bacterial diversity on different areas of broiler carcasses obtained at retail, using both classical isolations combined with MALDI-TOF MS identification and 16S amplicon sequencing. Furthermore, bacterial contaminations on poultry carcasses from the same and different processing plants are examined as a first assessment of the potential impact of poultry processing.

## Materials and Methods

### Sampling

From April to June 2018, 20 fresh, air packed broiler carcasses (sample information shown in Supplementary Table 1) were purchased 2–4 days before expiration day in supermarkets in Ghent, Belgium. Samples included eight organic-reared and 12 conventional-reared broilers processed in four different slaughterhouses (A–D), based on labels. Sampling was conducted within 1 h after purchase: 10 g neck skin, 10 g breast skin, and 10 g back skin were taken aseptically, and each homogenized separately with 1:10 (W/W) buffer peptone water (BPW, 3564684, Bio-RAD, France) in a sterile stomacher bag for 2 min at 230 rpm using a peristaltic homogenizer (Stomacher® 400 Circulator machine, Seward, UK).

### Bacterial Counts Examination

Ten-fold serial dilutions of each homogenate were prepared in BPW, followed by quantitative bacterial isolation in duplicate.

#### General Bacteria

Total aerobic bacteria isolation was performed on three non-selective media: plate count agar (PCA, 3564475, Bio-RAD) according to ISO 4833-2:2013 (ISO, 2013), trypticase soy agar (TSA, CM0131, OXOID, Basingstoke, UK), and blood agar (Columbia Agar Base, CM331, OXOID) with 5% defibrinated horse blood (E&O Laboratories Limited, Scotland) incubated either at 30°C for 72 h or at 7°C for 4–7 days.

Total anaerobic bacteria (TANAB) isolation was performed on PCA, TSA, and blood agar as described above, but incubated under anaerobic condition (BD GasPak™ EZ Anaerobe Container System, Becton, Dickinson and Company, United States) at 30°C for 48 h.

After incubation, colonies were counted from the plates with a bacterial growth between 30 and 300 CFU/plate.

#### Indicator Bacteria

Lactic acid bacteria (LAB) isolation was performed according to ISO 15214 on De Man, Rogosa, and Sharpe medium (MRS, 3564244, Bio-RAD) incubated at 30°C for 48 h anaerobically (ISO, 1998).

Presumptive *Pseudomonas* spp. were isolated according to ISO 13720 on cephalothin-sodium fusidate-cetrimide (CFC) agar with modified CFC selective supplement (CFC, CM0559B with SR0103E, OXOID), incubated at 30°C for 48 h aerobically (ISO, 2010).

*Escherichia coli* was selectively isolated on Tryptone Bile X-glucuronide (TBX, 3564035, Bio-RAD) incubated at 37°C for 24 h aerobically according to ISO 16649-2 (ISO, 2001).

Colonies were counted from the agar plates with a bacterial growth between 15 and 150 CFU/plate.

#### Foodborne Pathogens

*Campylobacter* isolation was performed according to ISO 10272-2: 1 ml of each neck, breast, and back skin homogenate was brought onto three modified *Campylobacter* blood-free selective agar (mCCDA) plates with CCDA selective supplement (CM0739 and SR0155H, OXOID) incubated at 41.5°C for 48 h micro-aerobically (ISO, 2017a). Suspected colonies were Gram-stained and further identified with MALDI-TOF MS analysis as described below.

*Salmonella* was isolated according to ISO 6579-1: the remaining homogenates were directly incubated at 37°C for 18 ± 2 h, after which 100 μl was dropped on modified semi-solid rappaport vassiliadis medium (MSRV, LAB150, LAB, UK) and incubated at 41.5°C for 24 ± 3 h (ISO, 2017b). Subcultures were taken from each outside edge of the halo and transferred onto xylose lysine deoxycholate agar (XLD, CM0469, OXOID) plates incubated at 37°C for 24 ± 3 h. Typical black colonies were picked and confirmed with MALDI-TOF MS analysis as described below.

### Homogenate and Plate Growth Profiling Using 16S rRNA Amplicon Sequencing

One carcass per rearing method and slaughterhouse was randomly chosen for subsequent bacterial community profiling (*n* = 5 out of 20; Supplementary Table 1). Of these carcasses, the skin homogenates were used directly for DNA extraction and sequencing. Also the bacterial growth of these homogenates on the aerobic counting plates was amplicon sequenced in parallel. The latter was done by collecting the biomass per plate (PCA, TSA, and blood agar) by plate washing technique as previously described (Yu et al., 2019). In brief, 15 ml homogenates (*n* = 15: three sampling areas from five carcasses) and 10 ml plate washings (*n* = 90: three general media at two temperatures from three sampling areas from five carcasses) were centrifuged at 8,000 *g* for 15 min and prepared for DNA extraction [DNeasy PowerFood Microbial Kit (Qiagen, Germany)] according to the manufacturer instructions. DNA quantity and quality were checked by Quantus™ Fluorometer (Promega, United States) and NanoDrop™ 2000/2000c Spectrophotometers (Thermo Fisher, United States), respectively. DNA samples were shipped to Novogene Institute (Hongkong, China) for 16S rRNA amplicon sequencing. The V3-V4 region of the 16S rRNA gene was amplified using primers 341F (CCTAYGGGRBGCASCAG) and 806R (GGACTACNNGGGTATCTAAT; Yu et al., 2005). DNA libraries of paired ends with single index were constructed using Truseq-dna-pcr-free-library-prep kit. Sequencing was performed on the Illumina Hiseq2500 platform. The raw data are available in the GenBank database under accession number SAMN15041177.

The QIIME2 (version 2018.06) software pipeline^1^ was used for data analysis. Reads were demultiplexed with q2-demux^2^. Then, the DADA2 plugin was implemented for the quality control process, and all phiX reads and chimeric sequences were filtered (Bokulich et al., 2017; Lee et al., 2019). Based on demux summary, sequences of both forward and reverse reads were truncated to a length with 154 bases. After denoising the data using the DADA2 denoise-paired method, representative sequences of each sample were retained and assigned to taxa using Naive Bayes classifiers pre-trained on Greengenes 13_8 99% OTUs full-length sequences^3^.

### MALDI-TOF MS Identification of Colonies

From the five carcasses selected for amplicon sequencing (Supplementary Table 1), a maximum of 60 colonies on the plates used for counting were randomly picked. They were subsequently pure-cultured on TSA and incubated at 30°C for 24 h. One colony of each pure-culture on the TSA plates was then picked using a sterilized toothpick and gently smeared onto a MALDI-TOF MS target plate (Bruker Daltonics, Bremen, Germany). After air-drying, the sample was covered with 1 μl matrix solution containing 10 mg/ml α-cyano-4-hydroxycinnamic acid in acetonitrile, deionized water, and trifluoracetic acid (50, 47.5, and 2.5 vv^−1^, respectively). Each series of measurements was preceded by a calibration step with a bacterial test standard (BTS 155 255343; Bruker Daltonics) to validate the run. Mass spectra were generated by a Micro-flex LT MALDI-TOF MS (Bruker Daltonics) equipped with a nitrogen laser (l1/4337 nm) operating in linear positive ion detection mode under Biotyper Automation Control 2.0 (Bruker Daltonics). Identifications were obtained by comparing the mass spectra to the Bruker MSP database (version DB5989) using the Bruker Compass software (Bruker Daltonics) at default settings. Identification at species or genus level was considered if scores were respectively above 2.0 and 1.7 according to the report generated by Bruker Compass.

Isolates for which no spectra or identification was obtained were analyzed again using the more extensive extraction method based on microorganism profiling “ethanol/formic acid extraction procedure” from Bruker Daltonics (03.04.2006). Briefly, a loop of bacteria was suspended in 300 μl milli-Q water and 900 μl ethanol, followed by centrifugation at 14,000 *g* for 3 min twice, the supernatant was discarded, and the ethanol-free pellet was suspended in 20–50 μl 70% formic acid, and equal volume of acetonitrile, followed by centrifugation at 14,000 *g* for 3 min. The supernatant was collected, and 1 μl was spotted in duplo on a MALDI-TOF MS target plate, covered by matrix solution, and measured as described above.

Isolates still without identification were subsequently analyzed by 16S rRNA gene sequencing. Therefore, one colony was suspended in 20 μl of lysis buffer (2.5 ml 10% SDS, 5 ml 1 N NaOH, and 92.5 ml Milli-Q water) and heated for 15 min at 95°C. After a short spin, 180 μl Milli-Q water was added. Subsequently, the supernatant was collected after centrifugation for 5 min at 10,000 *g* at 4°C. To amplify the partial 16S rRNA gene, the oligonucleotide primers pA (5'-AGA GTT TGA TCC TGG CTC AG-3') and pH (5'-AAG GAG GTG ATC CAG CCG CA-3') were used to amplify a 1.5 kb fragment (Edwards et al., 1989). The PCR mixture (final volume, 25 μl) contained 2.5 μl template DNA, 0.25 μl of each primer at a concentration of 10 μM, 2.5 μl of each deoxynucleoside triphosphate at a concentration of 2 μM each, 0.5 μl AmpliTaq DNA polymerase (1 U/μl), and 16.5 μl of Milli-Q water. The PCR protocol contained 30 cycles and was performed according to Edwards et al. (1989). Amplicons were collected and submitted for Sanger sequencing (Eurofins). Sequences were identified using BLAST^4^. For the taxa identification, the cut-off values for species at 98.65 and 94.5% for genus were applied (Kim et al., 2014; Yarza et al., 2014).

### Statistic Analysis

All enumeration data were checked for normality and homogeneity of variance using Shapiro-Wilk’s test and Levene’s test. Kruskal-Wallis H-test was used to assess significance of difference in bacterial levels on broiler carcasses between isolation media, incubation temperatures, sampling areas, rearing methods, and slaughterhouses. SPSS 20.0.0 was applied for these statistical analyses. Reported significant differences are corrected for multiple testing using Benjamini-Hochberg correction with 5% false discovery rate.

To identify differences in bacterial diversity between isolation media, sampling areas, rearing methods, and slaughterhouses, analysis of similarity (ANOSIM) was used to compare 16S amplicon sequencing data based on Bray-Curtis distance (Oksanen et al., 2008; Anderson and Walsh, 2013). Differences in abundance of each taxa between isolation media, sampling areas, rearing methods, and slaughterhouses were analyzed using DESeq (Love et al., 2014). Phyloseq was applied for analysis of alpha diversity and principal coordinate analysis (PCoA; McMurdie and Holmes, 2013). The significance of difference in bacterial richness between isolation media, sampling areas, rearing methods, and slaughterhouses was assessed using one-way ANOVA. All plots were generated using ggplot2 (Wickham, 2016) through R 3.6.1 (R Core Team, 2013).

## Results

### Culture Dependent Bacterial Contamination Determination

#### Comparison of Bacterial Counts on Different Sampling Area

**Figure 1:**
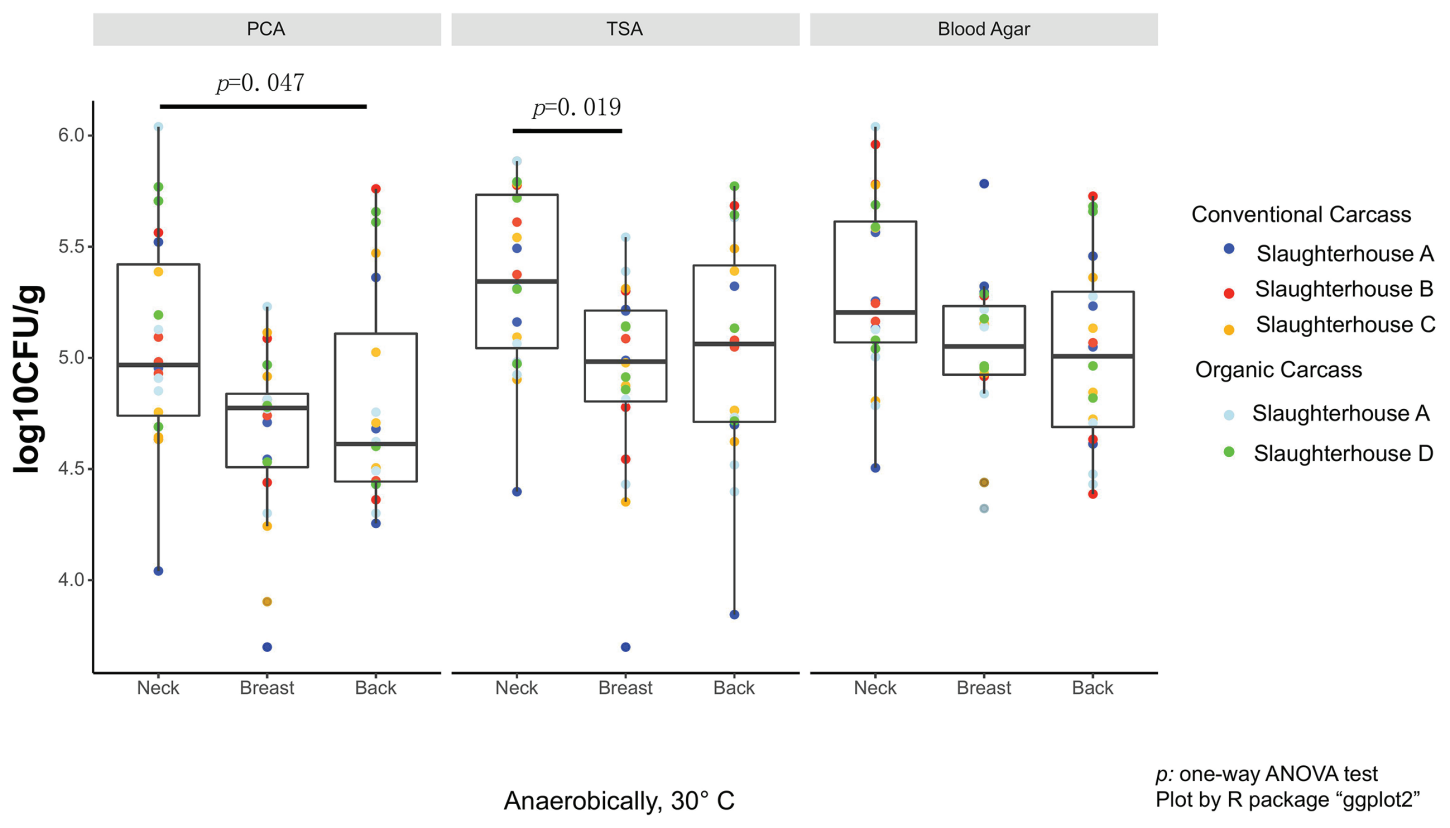
Total anaerobic bacteria (TANAB) counting on analysis plates from three sampling parts (neck, breast, and back skin).

As the data did not fall in normality distribution, non-parametric test (Kruskal-Wallis H-test) was used. On the anaerobic bacterial counts, using PCA plates, the neck skin (4.97 ± 0.68 log_10_ CFU/g, median ± IQR) was significantly more contaminated than the skin of the back area (4.67 ± 0.67 log_10_ CFU/g, *p* = 0.047; Figure 1), and also more, though not significant, than the breast skin (4.77 ± 0.33 log_10_ CFU/g). When TSA plates were used, the bacterial counts of the neck skin (5.34 ± 0.69 log_10_ CFU/g) were also significantly higher than the counts of the breast skin (4.98 ± 0.41 log_10_ CFU/g, *p* = 0.019), but not significantly different from the counts of the back skin (5.06 ± 0.70 log_10_ CFU/g). For the counts on blood agar, there was no significant difference in bacterial counts between the neck skin (5.20 ± 0.54 log_10_ CFU/g), breast skin (5.05 ± 0.31 log_10_ CFU/g), and back skin (5.01 ± 0.61 log_10_ CFU/g).

No overall differences were noted comparing all data of the total aerobic bacterial counts between the non-selective media (PCA, TSA, and blood agar) or between the two incubation temperatures (Supplementary Table 2 and Supplementary Figure 1).

Also, for the levels of indicator bacteria, presumptive *Pseudomonas* spp., LAB, and *E. coli*, between the sampling areas on the carcasses, no significant difference was observed (Supplementary Table 2).

#### Comparison of Bacterial Contamination Between Slaughterhouses

Between the four slaughterhouses, TAB counts determined on PCA and TSA plates at 7°C varied significantly. Considerable differences were observed between slaughterhouses B and C (PCA: *p* = 0.026; TSA: *p* = 0.039) and between slaughterhouses A and B (TSA: *p* = 0.028), where counts in samples from slaughterhouse B were significantly higher than those from A and C. None of the other general parameters (TANAB, TAB at 30°C) was slaughterhouse dependent (Supplementary Table 2).

For the indicator bacteria, the contamination levels were significantly different (Supplementary Table 2). The counts of presumptive *Pseudomonas* determined from samples of slaughterhouse B (5.06 ± 0.47 log_10_ CFU/g) were higher than from slaughterhouse A (4.62 ± 0.48 log_10_ CFU/g, *p* = 0.012). The counts of LAB from samples of slaughterhouse A (3.04 ± 1.25 log_10_ CFU/g) were significantly lower than in B (3.72 ± 1.35 log_10_ CFU/g, *p* = 0.011) and C (4.40 ± 0.73 log_10_ CFU/g, *p* = 3.5 × 10^−5^). The number of *E. coli* on the broiler carcasses was significantly higher in slaughterhouse D (4.15 ± 0.71 log_10_ CFU/g) compared to those in slaughterhouse A (3.34 ± 0.38 log_10_ CFU/g, *p* = 0.024), B (2.91 ± 0.85 log_10_ CFU/g, *p* = 0.012), and C (2.70 ± 0.20 log_10_ CFU/g, *p* = 1.0 × 10^−6^).

#### Impact of Rearing Method on Bacterial Contamination Levels

Comparing the results between organic‐ and conventional-reared animals, the aerobic bacteria counts determined on PCA plates at 30°C and the level of LAB were significantly higher (TAB: *p* = 0.04; LAB: *p* = 1.0 × 10^−6^) in the organically reared group (TAB: 4.94 ± 0.77 log_10_ CFU/g; LAB: 3.71 ± 0.74 log_10_ CFU/g) than in the conventional group (TAB: 4.83 ± 0.58 log_10_ CFU/g; LAB: 2.86 ± 0.72 log_10_ CFU/g). The level of presumptive *Pseudomonas* spp. was significantly higher (*p* = 0.01) in the conventional group (3.85 ± 1.01 log_10_ CFU/g) than in the organically reared group (3.01 ± 0.1.84 log_10_ CFU/g). All data are shown in Supplementary Table 2.

#### Pathogen Detection

*Salmonella* spp. were isolated from only 5% (1/20) of carcasses (conventionally reared), both on the neck and breast areas. One hundred percent of organic carcasses (8/8, slaughtered in A and D) and 8.33% of conventional-reared carcasses (1/12, slaughtered in A) were contaminated with *Campylobacter*. *Campylobacter* was present on all areas per carcass examined, with levels between 1 log_10_ CFU/g and 3 log_10_ CFU/g.

### Culture Dependent Microbial Identification Using MALDI-TOF MS

**Figure 2:**
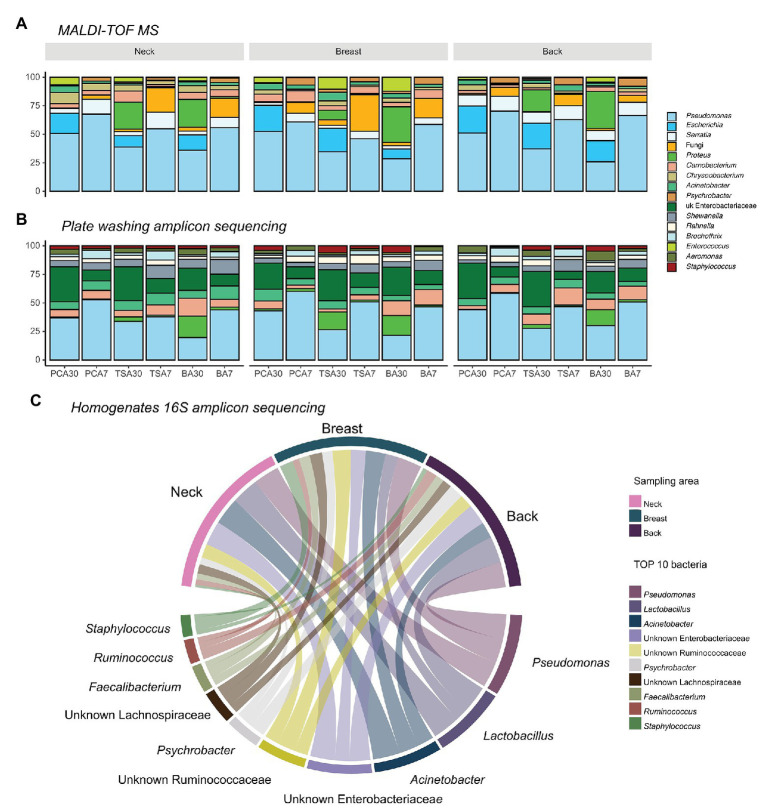
Top 10 abundant bacteria compositions on different sampling areas using different identification methods. **(A)** Top 10 bacteria on isolation plates examined using matrix-assisted laser desorption ionization time-of-flight mass spectrum (MALDI-TOF MS; 82.75%); **(B)** Top 10 bacteria on isolation plates examined using plate washing 16S amplicon sequencing (81.38%); and **(C)** top 10 abundant bacteria present in homogenates examined using 16S amplicon sequencing (65.31%).

In total, 5,172 isolates were harvested from PCA, TSA, and blood agar plates incubated at 30 and 7°C aerobically: 1,746 from the neck, 1,734 from the breast, and 1,692 from the back skin. *Pseudomonas* (*n* = 2,204) was always the most abundant genus present, regardless of the isolation medium applied, sampling area, or slaughterhouse (Figure 2). Two other commonly isolated bacteria were *Serratia* spp. (*n* = 344), which were mainly found in back samples (46.5%), but less in samples of the neck (33.7%) and the breast skin (19.8%), and *Escherichia* spp. (*n* = 392), which were mostly isolated from samples of the back (41.6%), breast (31.9%), and neck skin (26.5%).

Yeast and fungi (*n* = 343), including *Candida* spp., *Cryptococcus* spp., *Yarrowia* spp., and *Trichosporon* spp., were also present in samples of the breast (47.2%), neck (33.5%), and back skin (19.3%), and even more when an incubation temperature of 7°C was applied (87.5%). At this temperature, next to fungi, *Psychrobacter* spp. (*n* = 134) were the bacteria most commonly isolated (81.3%).

### Culture Independent Microbial Diversity Examination

In total, 60,868 and 280,668 reads were generated from the homogenates and plate washing DNA extracts, respectively. The reads in homogenates resulted in 1,187 amplicon sequence variants (ASVs) while 2,329 ASVs were retained in plate washings after non-bacterial reads filtered out using the quality-control plugin. Only ASVs accounting for more than 0.5% of the total reads were further retained (*n* = 53,472 reads in homogenates; *n* = 254,342 reads in plates washings). For the analyses, ASVs were summarized at genus-level and assigned at the lowest classified taxonomic rank, resulting in 35 taxonomic groups for the homogenates samples of which 29 could be assigned to genera, four to families, and two orders. Likewise, in the plate’s washings samples, 28 taxonomic groups were identified, of which 25 were assigned at genus, two at family, and one at order level.

#### Microbial Differences Between Isolation Plates Based on Amplicon Sequencing Data

The relative abundance of the genus *Proteus* was found to be higher on both TSA (*p* = 0.025) and blood agar (*p* = 0.0005) than PCA plates. No significant difference in abundance of taxa was observed between TSA and blood agar. When comparing bacterial richness, no significant difference between different isolation plates was observed.

Analysis of similarity revealed that dissimilarity of bacterial communities between plate types was not significantly different from the dissimilarity of communities within a plate type.

#### Compositional Differences Between Sampling Areas

When comparing the sequencing results of the homogenates and plates washing extracts, no difference in bacterial richness between neck, breast, and back skin was observed. The dissimilarity of bacterial communities between sampling areas was not significantly different from the dissimilarity of communities within an area, either. In Figure 2, the 10 most abundant bacteria in plate washings (81.38%; Figure 2B) and homogenates (65.31%; Figure 2C) are shown.

At genus level, *Pseudomonas*, *Acinetobacter*, *Lactobacillus*, unknown *Enterobacteriaceae*, unknown Ruminococcaceae, and *Psychrobacter* were most abundant in the homogenates. In homogenates of the neck skin, the top 50% bacteria comprised *Acinetobacter*, *Pseudomonas*, *Lactobacillus*, unknown *Enterobacteriaceae*, and unknown Ruminococcaceae, respectively. Similarly, in the homogenates of the breast skin, *Pseudomonas*, *Acinetobacter*, unknown Ruminococcaceae, unknown *Enterobacteriaceae*, *Staphylococcus*, unknown Lachnospiraceae, unknown bacteria, and *Faecalibacterium* were the top 50%. In the back-skin homogenates, the top 50% most abundant genera included *Pseudomonas*, *Lactobacillus*, unknown *Enterobacteriaceae*, *Acinetobacter*, *Psychrobacter*, and unknown Ruminococcaceae.

Analysis of the plate washing extracts revealed a significantly higher relative abundance of *Faecalibacterium* on the breast skin as compared to the neck (*p* = 1 × 10^−5^) and the back skin (*p* = 0.01). Furthermore, the relative abundance of unknown Clostridiales (*p* < 1 × 10^−8^), unknown *Desulfovibrionaceae* (*p* < 1 × 10^−8^), and *Kurthia* (*p* < 1 × 10^−8^) were found to be higher in the plate washing extracts of the neck skin than the breast skin.

#### Compositional Differences With and Without a Pre-cultivation

The bacterial richness was higher in the homogenates compared to the plates washing DNA extracts (*p* = 0.002). For the breast and back skin, significant differences in overall bacterial composition were found between homogenates and plate washings extracts, but not for the neck skin.

Overall, higher relative abundance of *Myroides* was found upon direct sequencing of the homogenates (*p* < 1 × 10^−8^). In the plate washing DNA extracts, the relative abundance of *Lactobacillus* (*p* = 0.013), *Faecalibacterium* (*p* = 0.019), *Psychrobacter* (*p* = 0.025), unknown Ruminococcaceae (*p* = 0.032), and *Acinetobacter* (*p* = 0.043) were significantly higher.

#### Compositional Differences Based on Slaughterhouse and Rearing Method

**Figure 3:**
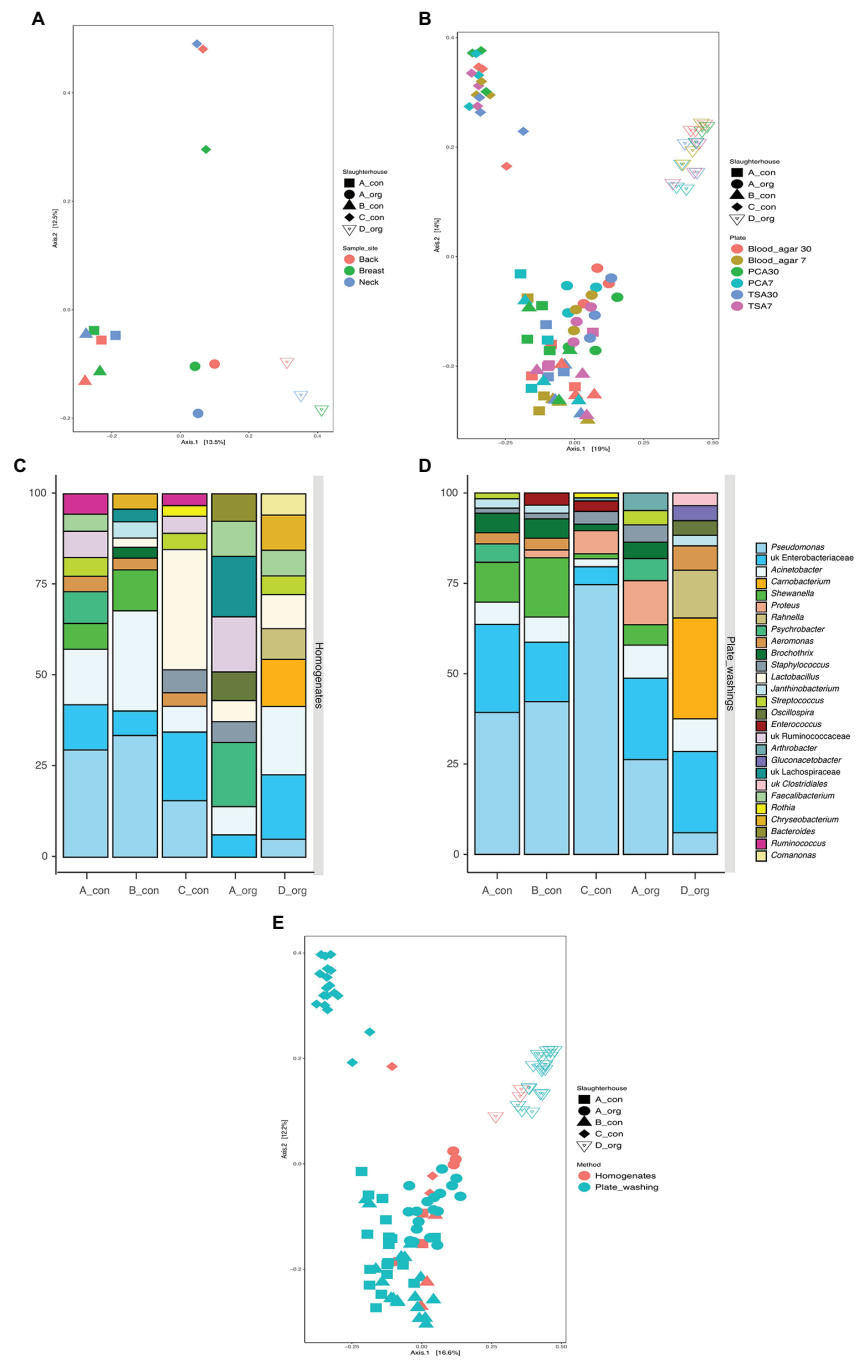
The similarity of bacterial communities between groups of samples. **(A,B)** Comparison of the influence of slaughterhouses (both), sampling areas **(A)**, and isolation plates **(B)**; **(C,D)** top 10 abundant bacteria on carcasses processed in each slaughterhouse using homogenates **(C)** and plates washing amplicon **(D)** sequencing (only top 10 taxa present in each bar); and **(E)** principal coordinate analysis (PCoA) for all samples examined using 16S amplicon sequencing.

Carcasses (*n* = 5) in the present study were obtained at retail, processed in four slaughterhouses, and reared either conventionally (*n* = 3) or biologically (*n* = 2). This offered the unique opportunity to explore potential slaughterhouse versus rearing method specific differences in microbiological communities. The PCoA analysis (Figure 3A) revealed that the different sampling sites clustered together per carcass. However, two conventionally reared chickens slaughtered in A and B shared a similar bacterial community, whereas all other carcasses displayed distinct microbial communities.

The bacterial richness was significantly higher in organic carcasses than conventional carcasses, and the bacterial communities between these carcasses were also significantly different. Amplicon sequencing directly on the homogenates revealed significantly higher relative abundance of *Lactobacillus* (*p* = 0.004) in conventional carcasses, whereas *Comamonas* (*p* = 0.005), *Carnobacterium* (*p* = 0.023), *Oscillospira* (*p* = 0.028), *Rahnella* (*p* = 0.036), *Ruminococcus* (*p* = 0.040), and *Staphylococcus* (*p* = 0.040) were significantly higher in organic carcasses. Moreover, the significantly different abundance between carcasses from different slaughterhouses examined using amplicon sequencing on the homogenates is shown in Table 1.

**Table 1:** The comparison of bacterial abundance between samples from different slaughterhouses (*p* < 0.05).

Slaughter-houses		Higher abundance			
Lower abundance		**A**	**B**	**C**	**D**
	**A**	X	*None*	^***^ *Lactobacillus*	
	**B**	^***^ *Lactobacillus/Staphylococcus/Comamonas/Ruminococcus/Bacteroides* ^**^ *Rahnella/Oscillospira/Faecalibacterium/Coprococcus/Carnobacterium* ^*^ *Clostridiale/Blautia/Streptococcus/Psychrobacter/*Ruminococcaceae	X	^***^ *Lactobacillus* ^**^ *Ruminococcus/Rahnella* ^*^ *Pseudomonas/*Enterobacteriaceae*/Acinetobacter/Shewanella*	^***^ *Lactobacillus/Comamonas/Rahnella* ^**^ *Ruminococcus/Carnobacterium/Bacteroides* ^*^ *Faecalibacterium/Proteus/Streptococcus*
	**C**	^***^ *Psychrobacter/Staphylococcu/Comamonas/Bacteroides* ^**^ *Ruminococcus/Acinetobacte/Coprococcus* ^*^ *Shewanella*	^***^ *Psychrobacter* ^*^ *Acinetobacter/Shewanella*	X	^***^ *Psychrobacter/Comamonas/Staphylococcus* ^**^ *Bacteroides* ^*^ *Acinetobacte/Shewanella*
	**D**	^***^ *Staphylococcus* ^**^Ruminococcaceae ^*^ *Coprococcus*	^***^ *Staphylococcus*	^**^Ruminococcacea*/Lactobacillus*	X

* *p* < 0.05;

** *p* < 0.01;

*** *p* < 0.00001.

On the other hand, after cultivation and plate washing extract sequencing, the relative abundance of *Carnobacterium* (*p* < 1 × 10^−8^), unknown Enterobacteriaceae (*p* < 1 × 10^−8^), *Rahnella* (*p* < 1 × 10^−8^), *Acinetobacter* (*p* < 1 × 10^−8^), *Aeromonas* (*p* = 1 × 10^−5^), *Staphylococcus* (*p* = 0.0001), and *Proteus* (*p* = 0.001) was significantly higher in organic carcasses (Supplementary Figure 2).

## Discussion

In the present study, the contamination levels as well as the bacterial diversity on different areas of 20 broiler carcasses obtained at retail were determined using both classical isolations combined with MALDI-TOF MS identification and 16S amplicon sequencing.

Isolation media, incubation temperature, and time all determine the final outcome of a bacteriological examination of food and can bias extrapolation and comparison of data between studies. No significant differences were, however, recorded, pointing that the impact of general isolation media and incubation temperature, with the exception of a longer incubation time, is negligible when only quantitative and superficial taxonomic level data are required. In the current study, cultivation combined with identification provided just as accurate relevant information as when culture independent methods were applied, including levels and abundance of most relevant spoilage bacteria.

In general, as shown in Figure 2, the most abundant genus recovered using classical isolation combined with MALDI-TOF MS identification was *Pseudomonas* independent of the isolation medium applied, sampling area, or slaughterhouse. This finding was confirmed in the sequencing analyses, where *Pseudomonas* was always among the most abundant genera in the homogenates. Based on the culture-dependent analyses, two other genera were commonly isolated, *Serratia* spp. and *Escherichia* spp. The high abundance of unknown Enterobacteriaceae might comprise *Escherichia* spp. and *Serratia* spp. in the sequencing analyses. Vice versa, using sequencing analyses, some additional abundant genera were detected that were not recovered with culture-based isolation methods, underscoring the complementarity of both approaches. Interestingly, bacterial richness was higher in the homogenates compared to the plate washing DNA extracts, meaning that pre-cultivation had a significant impact on the observed bacterial richness and thereby skews the results. The plate washings sequencing reflects the cultivable communities on food samples, which are more studied in routine food microbiology examination. Interestingly, the bacterial communities on the neck skin had no significant difference between homogenates and plate washings, suggesting that applying amplicon sequencing on the neck skin has limited added value. However, amplicon sequencing on the back or breast skin samples offers a novel perspective on the bacterial community and allows the detection of spoilage bacteria, other than *Pseudomonas*.

In the present study, the culture independent microbial diversity analyses were done on five carcasses that had been slaughtered in these four slaughterhouses. The PCoA analysis (Figure 3A) suggested a bird or a slaughterhouse depending microbial composition as different sampling areas did not cluster together per carcass. However, two conventionally reared chickens slaughtered in A and B shared a similar bacterial community, whereas all other carcasses displayed distinct microbial communities. Taken together the culture-dependent results strongly suggest that the slaughterhouse environment affects the contamination on the carcasses while the sequencing results do not allow exclusion of an effect of the initial chicken microbiota (Figures 3B,E). The LAB levels were significantly different between carcasses from different slaughterhouses. In-depth analyses revealed that the carcass slaughtered in C was mostly predominant by *Lactobacillus* rather than *Pseudomonas* (Figures 3C,D). In a previous study, the defeathering time was found related to the lactic acid bacterial level (Cason et al., 2004). Although water of drinking quality is obligatorily applied for scalding and (final) rinsing, the water pH is different between geographical areas (Olayinka et al., 2013) and might have an impact on the LAB level. This suggests that the slaughterhouse environment might add a specific bacterial population to the processed meat.

The present study also aimed to assess, as a first exploration, differences between poultry rearing systems. Overall, the organically reared chickens had a richer microbial profile as compared to the conventional reared chickens. Further analysis revealed that they also displayed a distinct microbial signature; however, this signature changed upon pre-cultivation but remained different from conventionally reared chickens. The latter suggest that the cultivable fraction on both types of chickens was significantly different, which can have an impact on spoilage rates. The more variable bacterial communities on organical carcasses compared to the conventional ones might be related to the rearing farms. The aspects of organic farms are outdoor rearing, inducing more contacts with natural contaminants, such as soil and insects. The analyses also suggested an impact of rearing methods on bacterial contamination levels as the aerobic bacteria counts were significantly higher in the organically reared group while the level of presumptive *Pseudomonas* spp. was significantly higher in the conventional group. The PCoA plot based on the sequencing results also suggests that the rearing method might overrule chicken or slaughterhouse specific signature as samples from organically reared chicken are located separate from the rest of the samples (Figure 3A). Equipment, staff, and origin of the birds in each slaughterhouse could shape a unique bacterial community on broiler carcasses (Tsola et al., 2008; Fallschissel et al., 2010). These findings suggest that both farm and slaughterhouse can affect the initial bacterial community on broiler carcasses.

The present study also examined bacterial contamination level and diversity on different areas of broiler carcasses. As already commonly accepted, the neck skin turned out again to be the highest contaminated area, both with aerobic and anaerobic bacteria, followed by the back and then the breast area. In previous studies, some hypotheses have been proposed to explain this observation. Lillard et al. (1986) proposed that bacteria might attach more firmly to follicles, which are more present on the neck skin area. Other studies state that the viable bacterial contamination level has no significant correlation with the density of feather follicles but is related to fecal contamination during slaughter, in particular with content of the large intestine (Buhr et al., 2003; Cason et al., 2004). In line with the latter statement, in the present study, the counts of anaerobic bacteria, which are more abundant in the cecum and ileum content, were significantly higher on the neck skin (Lu et al., 2003). Due to the “head down” position (Wu et al., 2014), the neck area also collects more contaminated process water in particular during scalding, in which high levels of intestinal associated anaerobic and faculty anaerobic bacteria are present (Delforno et al., 2017). *Lactobacilli*, present on the skin but predominant in the ileac content and fecal matter of broilers (Barnes, 1979; Lu et al., 2003; Hinton and Cason, 2008), were present as top 50% taxa on the neck and back areas rather than on the breast area. After cultivation, the abundance of unknown Clostridiales, which is more abundant in cecum content (Stanley et al., 2015), was significantly higher on the neck skin. After evisceration, the (ruptured) intestinal tract can be placed on the backside, where the intestinal content can contaminate the carcasses. It suggests again contamination from the intestinal content to the skin, as discussed above. However, besides fecal contamination during slaughtering, the breast skin could also have been contaminated during rearing at the farm. The breast area of industrial broilers is usually less feathered due to the intensive rearing system, and this exposes the birds more directly to feces and spilled feed. *Faecalibacterium* spp., commonly present in both cecal and fecal content, were significantly more present on the breast skin, indicating that this sampling area may be preferential for bacteria due to different exposures to the environment. The different abundances of bacterial populations between sampling areas suggests that spoilage rates between these areas could be different. Although *Pseudomonas* is considered as the main indicator for spoilage, and was also most abundant over other spoilage related genera, other bacteria also determine the spoilage rate. For example, *lactobacilli*, of which the relative abundance was higher on the neck and back skin, could induce odor through lactic acid production earlier than the breast skin (Figure 2C).

In conclusion, PCA plates incubated at 30°C were the best fit for viable aerobic bacterial counts on broiler carcasses. Compared to the breast skin, the neck, and back skin tend to spoil faster than the breast skin when anaerobic storage conditions are applied. The distribution of bacteria in different areas can also be related to potential contamination source during the slaughter process. Bird-rearing method and slaughterhouses may have an impact on final bacterial contamination of broiler carcasses, but a further correlation between bacterial contamination on carcasses at retail and the potential factors in different bird-rearing methods, as well as the impact of slaughterhouses, should be assessed.

## Data Availability Statement

The original contributions presented in the study are publicly available. This data can be found here: https://www.ncbi.nlm.nih.gov/, with accession number PRJNA635448.

## Author Contributions

ZY and KH designed the study. ZY performed the experiments and analyzed the data. ZY, KH, and MJ compiled the manuscript. All authors contributed to the article and approved the submitted version.

### Conflict of Interest

The authors declare that the research was conducted in the absence of any commercial or financial relationships that could be construed as a potential conflict of interest.

## Footnotes

^1^https://qiime2.org

^2^https://github.com/qiime2/q2-demux

^3^https://docs.qiime2.org/2017.12/data-resources/

^4^https://www.ncbi.nlm.nih.gov
