# Supplementary material for: Analyses of the Bacterial Contamination on Belgian Broiler Carcasses at Retail Level
Source: Front Microbiol. 2020 Sep 16;11:539540. doi: 10.3389/fmicb.2020.539540 (PMC7525026; doi:10.3389/fmicb.2020.539540)
Supplement: Supplementary file 1 [file Data_Sheet_1.docx]

Supplementary Material

| Chicken carcass | Slaughterhouse | Organic | Bacterial counts | Bacterial diversity | | |
| --- | --- | --- | --- | --- | --- | --- |
|  |  |  |  | MALDI-TOF MS | SH amplicon sequencing | PW amplicon sequencing  (PCA,TSA, and Blood agar at 30°C and 7°C) |
| 1 | A | \ | Yes | \ | \ | \ |
| 2 | A | \ | Yes | Yes | Yes | Yes |
| 3 | A | \ | Yes | \ | \ | \ |
| 4 | A | \ | Yes | \ | \ | \ |
| 5 | B | \ | Yes | \ | \ | \ |
| 6 | B | \ | Yes | \ | \ | \ |
| 7 | B | \ | Yes | \ | \ | \ |
| 8 | B | \ | Yes | Yes | Yes | Yes |
| 9 | C | \ | Yes | Yes | Yes | Yes |
| 10 | C | \ | Yes | \ | \ | \ |
| 11 | C | \ | Yes | \ | \ | \ |
| 12 | C | \ | Yes | \ | \ | \ |
| 13 | A | Yes | Yes | \ | \ | \ |
| 14 | A | Yes | Yes | \ | \ | \ |
| 15 | A | Yes | Yes | \ | \ | \ |
| 16 | A | Yes | Yes | Yes | Yes | Yes |
| 17 | D | Yes | Yes | Yes | Yes | Yes |
| 18 | D | Yes | Yes | \ | \ | \ |
| 19 | D | Yes | Yes | \ | \ | \ |
| 20 | D | Yes | Yes | \ | \ | \ |

Supplementary Table1 Sampling information

Supplementary Table2. Quantitative analysis result of general bacteria, specific bacteria and pathogen (lg CFU/g).

| Slaughterhouses | Organic | Sampling site | PCA | | | | | | TSA | | | | | | Blood Agar | | | | | | CFC | | MRS | | TBX | |
| --- | --- | --- | --- | --- | --- | --- | --- | --- | --- | --- | --- | --- | --- | --- | --- | --- | --- | --- | --- | --- | --- | --- | --- | --- | --- | --- |
|  |  |  | 30°C | | 7°C | | Anaerobic | | 30°C | | 7°C | | Anaerobic | | 30°C | | 7°C | | Anaerobic | |  |  |  |  |  |  |
|  |  |  | Median | IQR | Median | IQR | Median | IQR | Median | IQR | Median | IQR | Median | IQR | Median | IQR | Median | IQR | Median | IQR | Median | IQR | Median | IQR | Median | IQR |
| A | N | Neck | 4.94 | 0.82 | 4.84 | 0.45 | 4.79 | 0.61 | 5.20 | 0.74 | 4.52 | 1.09 | 5.24 | 0.39 | 5.07 | 1.06 | 4.92 | 0.45 | 5.19 | 0.36 | 4.33 | 0.94 | 3.72 | 0.24 | 3.39 | 0.67 |
|  |  | Breast | 4.94 | 0.61 | 4.90 | 0.71 | 4.63 | 0.40 | 5.03 | 0.78 | 4.63 | 0.77 | 5.10 | 0.55 | 4.93 | 0.63 | 4.82 | 0.47 | 5.31 | 0.36 | 4.42 | 0.42 | 3.85 | 0.39 | 2.70 | 0.69 |
|  |  | Back | 4.77 | 0.46 | 4.56 | 0.69 | 4.56 | 0.46 | 4.91 | 0.61 | 4.74 | 0.62 | 4.89 | 0.65 | 4.75 | 0.57 | 4.63 | 0.88 | 5.14 | 0.35 | 3.87 | 0.69 | 3.02 | 0.18 | 3.13 | 1.01 |
| B | N | Neck | 5.38 | 0.14 | 5.23 | 0.29 | 5.04 | 0.24 | 5.73 | 0.14 | 5.49 | 0.26 | 5.69 | 0.25 | 5.64 | 0.15 | 5.42 | 0.38 | 5.51 | 0.60 | 5.38 | 0.47 | 4.67 | 0.79 | 3.26 | 0.77 |
|  |  | Breast | 4.75 | 0.09 | 5.05 | 0.16 | 4.78 | 0.22 | 5.17 | 0.13 | 5.36 | 0.17 | 4.93 | 0.42 | 5.16 | 0.07 | 5.21 | 0.14 | 5.04 | 0.26 | 4.85 | 0.05 | 3.72 | 0.50 | 3.03 | 0.62 |
|  |  | Back | 5.43 | 0.26 | 5.28 | 0.32 | 4.48 | 0.39 | 5.56 | 0.43 | 5.33 | 0.14 | 5.06 | 0.25 | 5.44 | 0.52 | 5.26 | 0.36 | 4.85 | 0.66 | 5.20 | 0.28 | 3.48 | 0.82 | 2.66 | 0.73 |
| C | N | Neck | 4.84 | 0.29 | 4.70 | 0.36 | 4.70 | 0.27 | 5.14 | 0.31 | 5.10 | 0.52 | 5.32 | 0.56 | 5.06 | 0.29 | 5.02 | 0.26 | 5.33 | 0.62 | 4.92 | 0.43 | 4.90 | 0.46 | 2.65 | 0.17 |
|  |  | Breast | 4.45 | 0.48 | 4.12 | 0.49 | 4.58 | 0.81 | 4.60 | 0.69 | 4.37 | 0.38 | 4.93 | 0.32 | 4.66 | 0.66 | 4.35 | 0.31 | 4.94 | 0.20 | 4.06 | 0.37 | 4.06 | 0.65 | 1.50 | 3.02 |
|  |  | Back | 4.64 | 0.39 | 4.55 | 0.49 | 4.87 | 0.48 | 4.74 | 0.37 | 4.80 | 0.60 | 5.08 | 0.69 | 4.86 | 0.24 | 4.74 | 0.42 | 4.99 | 0.38 | 4.56 | 0.70 | 4.18 | 0.34 | 2.74 | 0.27 |
| A | Y | Neck | 4.87 | 0.35 | 5.01 | 0.49 | 5.02 | 0.46 | 5.14 | 0.40 | 5.11 | 0.40 | 5.02 | 0.30 | 4.98 | 0.39 | 4.98 | 0.43 | 5.07 | 0.41 | 4.76 | 0.41 | 2.39 | 0.75 | 3.41 | 0.28 |
|  |  | Breast | 5.06 | 0.74 | 5.08 | 0.57 | 4.79 | 0.26 | 5.01 | 0.71 | 5.21 | 0.64 | 5.10 | 0.71 | 5.00 | 0.76 | 5.06 | 0.64 | 4.99 | 0.45 | 4.76 | 0.43 | 1.20 | 2.59 | 3.31 | 0.11 |
|  |  | Back | 4.86 | 0.36 | 4.80 | 0.57 | 4.56 | 0.21 | 4.87 | 0.42 | 4.88 | 0.62 | 4.63 | 0.47 | 4.76 | 0.45 | 4.83 | 0.44 | 4.59 | 0.38 | 4.64 | 0.48 | 2.39 | 0.09 | 3.56 | 0.22 |
| D | Y | Neck | 5.41 | 0.74 | 5.04 | 0.95 | 5.45 | 0.65 | 5.57 | 0.50 | 5.12 | 0.83 | 5.51 | 0.51 | 5.51 | 0.57 | 4.98 | 0.91 | 5.33 | 0.54 | 4.95 | 0.58 | 4.18 | 0.46 | 4.17 | 0.59 |
|  |  | Breast | 4.92 | 0.23 | 4.41 | 0.28 | 4.78 | 0.11 | 5.26 | 0.18 | 4.60 | 0.23 | 5.03 | 0.24 | 5.17 | 0.17 | 4.60 | 0.23 | 5.07 | 0.24 | 4.10 | 0.33 | 3.32 | 2.63 | 4.37 | 0.64 |
|  |  | Back | 5.29 | 0.63 | 5.33 | 0.82 | 5.11 | 1.06 | 5.23 | 0.25 | 5.36 | 0.92 | 5.39 | 0.65 | 5.42 | 0.48 | 5.19 | 0.61 | 5.31 | 0.74 | 4.98 | 0.90 | 4.31 | 0.74 | 4.08 | 0.60 |

Supplementary Figure 1 Total viable bacteria counting on analysis plates from three sampling part (neck, breast and back skin). Arrows in same color indicate the significant difference between 2 sampling areas under the same incubation condition (isolation plates, temperature and aerobic atmosphere).


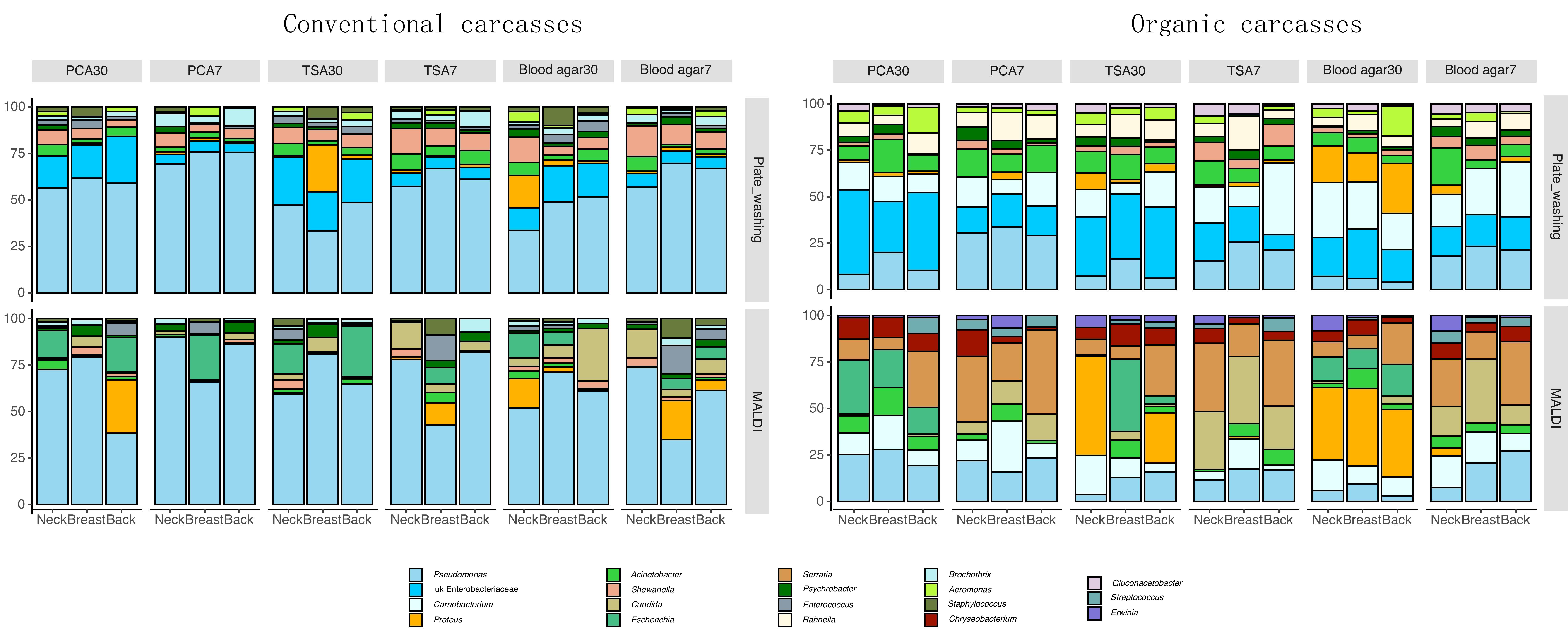
Supplementary Figure 2 Bacterial communities between conventional carcasses and organic carcasses identified after cultivation.
